# Is Intestinal Microbiota Fully Restored After Chickens Have Recovered from Coccidiosis?

**DOI:** 10.3390/pathogens14010081

**Published:** 2025-01-16

**Authors:** Jiaqing Guo, Zijun Zhao, Chace Broadwater, Isabel Tobin, Jing Liu, Melanie Whitmore, Guolong Zhang

**Affiliations:** Department of Animal and Food Sciences, Oklahoma State University, Stillwater, OK 74078, USA; jiaqing.guo@okstate.edu (J.G.); zijun.zhao@okstate.edu (Z.Z.); chace.mccoy@okstate.edu (C.B.); isabel.tobin@okstate.edu (I.T.); jing.liu12@okstate.edu (J.L.); melanie.whitmore@okstate.edu (M.W.)

**Keywords:** microbiome, *Eimeria*, 16S rRNA gene sequencing, lactic acid bacteria, pathobionts, short-chain fatty acid-producing bacteria, probiotics

## Abstract

The intestinal microbiota is known to be altered by *Eimeria*-induced coccidiosis, but it remains unclear whether the microbiota is fully restored after recovery. To address this, 110 newly hatched Cobb male broiler chickens were challenged with 2 × 10^4^ sporulated oocysts of *Eimeria maxima* (EM) strain M6 or mock-infected with saline on day 10. Body weight and feed intake were recorded. Additionally, 10 mock- and 12 EM-infected birds were randomly selected to assess the small intestinal lesion, fecal oocyst shedding, and ileal and cecal microbiota compositions using 16S rRNA gene sequencing at 3, 5, 7, 14, and 21 days post-infection (dpi). EM infection significantly decreased (*p* < 0.001) body weight by 5 dpi, persisting through 21 dpi. The infection also reduced (*p* < 0.05) weight gain, feed intake, and feed efficiency in the first week; however, these parameters became comparable in the second and third weeks. At 7 dpi, during the peak of infection, major lactic acid bacteria were enriched, while short-chain fatty acid-producing bacteria were mostly suppressed in both the ileum and cecum. Opportunistic pathogens such as *Escherichia* and *Clostridium perfringens* transiently bloomed at 7 dpi. By 14 dpi, differential bacterial enrichment subsided, and nearly all commensal bacteria returned to healthy levels by 21 dpi. Coupled with comparable growth performance between healthy and EM-recovered chickens, we conclude that the intestinal microbiota is largely restored to its healthy state after recovery. Understanding the microbiota’s responses to coccidiosis may inform probiotic-based mitigation strategies.

## 1. Introduction

The stability of the intestinal microbiota is crucial for maintaining the host’s normal physiological and immune functions [1,2]. While the microbiome can be disrupted by various factors, such as environmental changes, diet, antibiotics, stress, and infections, it often shows a remarkable ability to recover and return to its original healthy state after the disturbance is removed [3]. However, some disturbances may lead the microbiome to an alternative stable healthy state rather than its original state [3]. For example, antibiotic therapy typically results in an initial decrease in bacterial diversity, followed by a partial return to a composition similar to the initial state, though full recovery is rarely achieved [4]. Similarly, children recovering from severe acute malnutrition often have a disrupted intestinal microbiome that may not be fully restored to a healthy state even with nutritional rehabilitation, affecting their long-term growth and development [5]. Additionally, some patients recovering from COVID-19 still exhibit significant differences in their intestinal microbiota compared to healthy individuals three months post-recovery [6].

Coccidiosis, caused by the genus *Eimeria*, is a prevalent enteric disease in poultry [7,8]. These parasites invade and proliferate within intestinal epithelial cells after the ingestion of infective sporulated oocysts by chickens, undergoing several rounds of asexual and sexual reproduction [7,8]. This process damages the intestinal mucosal surface, triggers inflammation, compromises nutrient digestion and absorption, and reduces growth performance. Clinical symptoms include bloody diarrhea, dehydration, loss of appetite, and lethargy. Although coccidiosis is not typically fatal, it results in significant economic losses, costing the global poultry industry approximately USD 13 billion annually due to reduced production efficiency [9]. *Eimeria maxima* (EM) primarily infects and damages the jejunum and ileum of chickens, completing its life cycle in 6–7 days and causing maximum damage to the intestinal mucosal surface by 7 days post-infection (dpi) [10].

A typical course of EM-induced coccidiosis consists of the prepatent, acute, and recovery phases [7,8]. The prepatent phase occurs during the initial 3–4 days of infection without intestinal damage or clinical symptoms. The acute phase, between 5 and 7 dpi, is characterized by damage to the intestinal mucosal epithelia and associated clinical symptoms. Following the release of oocysts from intestinal epithelial cells at 7 dpi, mucosal damage and clinical symptoms begin to resolve, and animal growth performance recovers unless a new round of infection occurs.

It is well documented that the intestinal microbiota is altered in response to *Eimeria* infection [11,12]. However, it remains unclear whether the microbiome returns to its original state or shifts to an alternative healthy state after recovery from coccidiosis. Previous studies have examined bacterial changes in response to *Eimeria* infection up to 14 dpi [13,14], indicating a tendency for the intestinal microbiota to return to normal. However, the microbiome composition is not fully restored, likely due to the short recovery period of one week. To gain a better understanding of the potential complete restoration of the intestinal microbiota, we extended the observation period to 21 dpi, allowing for two weeks of recovery from coccidiosis beyond the acute phase.

## 2. Materials and Methods

### 2.1. Chicken Model of Coccidiosis

A total of 110 newly hatched, unvaccinated male Cobb broiler chicks were obtained from the Cobb-Vantress Hatchery (Siloam Springs, AR, USA) and housed in floor pens covered with fresh pinewood shavings in an environmentally controlled room under standard management. Chicks were randomly allocated to six pens, each containing 16 to 20 birds, with free access to tap water and a mash corn–soybean meal standard starter diet (21% crude protein) throughout the trial. Coccidiosis was induced as described [15]. Briefly, 10-day-old chicks in three pens were inoculated orally with 2 × 10^4^ sporulated oocysts of *E. maxima* strain M6 (kindly provided by John R. Barta, University of Guelph, Canada) in 1 mL saline after overnight fasting. The remaining chickens were mock-infected with 1 mL saline. The pens for the two groups of chickens were separated with plastic sheets, and extreme cautions were taken to minimize cross-contamination during animal care and sampling. 

Animals were observed daily for clinical signs of coccidiosis, such as lethargy, bloody diarrhea, and ruffled feathers. Wood shavings were replaced with fresh shavings at 8 dpi to minimize parasite recycling. All animals were weighed individually, and feed intake was also recorded by pen at 3, 5, 7, 14, and 21 dpi. At each time point, 10 birds from the mock-infected group and 12 birds from the infected group were randomly selected and euthanized via CO_2_ asphyxiation. Approximately 0.5 g of proximal ileum digesta and approximately 0.2 g of cecal digesta were squeezed aseptically from each animal, snap-frozen in liquid nitrogen, and stored at −80 °C until further analysis. The remaining jejunum and ileum were cut open and scored for gross lesions using a 4-point scoring system [16]. The average daily weight gain, daily feed intake, and feed conversion ratio (feed intake/weight gain) were calculated on a weekly basis following infection. All animal procedures were approved by the Institutional Animal Care and Use Committee (IACUC) of Oklahoma State University under protocol number AG-21–62.

### 2.2. Bacterial DNA Isolation and 16S rRNA Gene Sequencing

Bacterial DNA from ileal and cecal digesta samples was isolated using Fecal DNA MicroPrep and MiniPrep Kits (Zymo Research, Irvine, CA, USA), respectively. DNA concentration and quality were assessed using a Nanodrop One Spectrophotometer (Thermo Fisher Scientific, Waltham, MA, USA). High-quality DNA samples were sent to Novogene (Beijing, China) for PCR amplification of the V3-V4 region of the bacterial 16S rRNA gene using primers 341F (CCT AYG GGR BGC ASC AG) and 806R (GGA CTA CNN GGG TAT CTA AT). The amplicons were sequenced (2 × 250 bp paired-end) on an Illumina NovaSeq 6000. 

### 2.3. Bioinformatics

Downstream bioinformatic analysis was conducted as we previously described [14,17]. Briefly, raw sequencing reads were processed with QIIME2 v2023.9 [18]. The reads were joined using “vsearch”, followed by quality control using “quality-filter” and denoising using “Deblur”, resulting in 402-nucleotide amplicon sequence variants (ASVs). ASVs were classified using a naïve Bayesian classifier against the RDP classifier 2.14 (August 2023) [19] with 97% confidence. The top 100 ASVs were further reclassified and confirmed using the EzBioCloud 16S database (2023.08.23) [20] with 97% identity. Further downstream analysis and visualization were performed in R (v4.4.1) using ASVs present in >5% of samples. Rarefied read counts were used in ‘phyloseq’ v1.48.0 [21] to calculate α-diversity (including the number of ASVs, Pielou’s Evenness, and Shannon index) and β-diversity (including the weighted and unweighted UniFrac distances). Differential abundance analysis was performed using raw read counts in ANCOM-BC v2.6.0 [22]. 

### 2.4. Statitical Analysis

Statistical significance between the EM- and mock-infected groups was assessed using Student’s *t*-test when the normality and homogeneity of variance of the data were confirmed by the Shapiro–Wilk test and Levene’s test, respectively. Otherwise, the Mann–Whitney U test was used. The Kruskal–Wallis test and post hoc Dunn’s test were applied to compare intestinal lesion scores at different times after infection. The β-diversity between the two groups was compared using permutational multivariate analysis of variance (PERMANOVA) with 999 permutations, implemented in the ‘vegan’ package (v2.6-8). A *p*-value or adjusted *p*-value (if applicable) of < 0.05 was considered statistically significant.

## 3. Results

### 3.1. Growth and Pathological Response to E. maxima Infection

**Figure 1:**
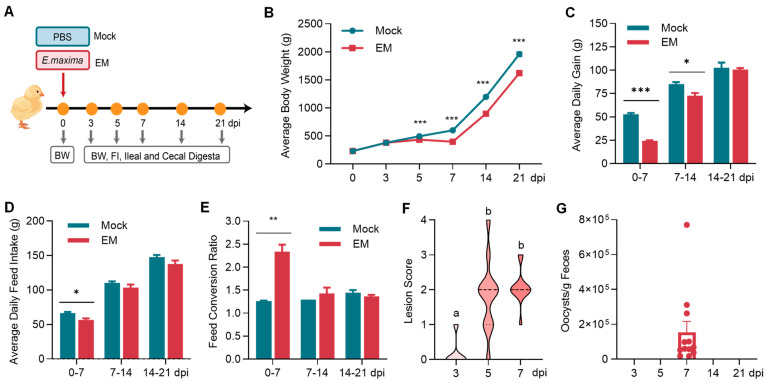
Growth performance, intestinal lesion, and oocyst shedding of the chickens in response to *Eimeria* infection. (**A**) Experimental scheme. Day-10 Cobb chicks were weighed individually and inoculated orally with saline (mock) or 2 × 10^4^ sporulated oocysts of *E. maxima* strain M6 (EM), respectively. Body weight (BW) and feed intake (FI) were recorded at 3, 5, 7, 14, and 21 days post-infection (dpi). At each time point, 10 and 12 chicks were randomly selected from the mock and EM groups, respectively, and the ileal and cecal digesta were collected from each animal. (**B**) Average body weight, (**C**) Average daily gain, (**D**) average daily feed intake, and (**E**) feed conversion ratio of individual animals at different times post-infection. (**F**) Small intestinal lesion scores of EM-infected chickens. (**G**) Fecal shedding of oocysts in EM-infected chickens. The data are presented as means ± SEM. Student’s *t*-test was performed in panels (**B**–**E**,**G**), with * *p* < 0.05, ** *p* < 0.01 and *** *p* < 0.001. The Kruskal–Wallis test and post hoc Dunn’s test were used in panel F, with different letters indicating *p* < 0.05.

To investigate if the intestinal microbiota is fully restored to normal after recovery from coccidiosis, Cobb chickens were inoculated with either saline or EM on day 10, their body weight and feed intake were recorded, and the ileal and cecal digesta were collected for microbiome analysis at 3, 5, 7, 14, and 21 dpi (Figure 1A). As expected, despite the lack of mortalities throughout the trial, EM-infected chicks exhibited significant growth retardation starting at 5 dpi, which persisted through 21 dpi (*p* < 0.001) (Figure 1B). During the first week of infection, growth retardation was approximately 50% (24.2 g vs. 52.8 g daily gain in EM- and mock-infected groups, respectively). This condition improved in the second week, with no significant difference observed in the third week between the two groups, indicating recovery from coccidiosis, albeit without compensatory growth (Figure 1C).

Similarly, feed intake was significantly reduced during the first week of infection (*p* < 0.05) but largely recovered during the second and third weeks (Figure 1D). Consequently, the feed conversion ratio was significantly higher in EM-infected chickens compared to mock-infected chickens during the first week (*p* < 0.01) but was comparable between the two groups in the second and third weeks (Figure 1E). As expected, small intestinal lesions became more pronounced over time, peaking at 7 dpi before fully recovering (Figure 1F). As expected, oocyst shedding in the feces was observed only at 7 dpi (Figure 1G), aligning with the life cycle of *E. maxima*.

### 3.2. Ileal Microbiome Dynamics in Response to E. maxima Infection

To monitor the dynamic changes in the intestinal microbiota following EM infection, ileal and cecal digesta samples were collected at 3, 5, 7, 14, and 21 dpi and subjected to 16S rRNA gene sequencing. After primer removal, joining of paired reads, and quality control, a total of 13,587,920 high-quality sequences were obtained from 107 ileal and 107 cecal samples, averaging 63,495 ± 5914 sequences per sample. After denoising and filtering out ASVs present in < 5% of samples, 470 ASVs were retained.

**Figure 2:**
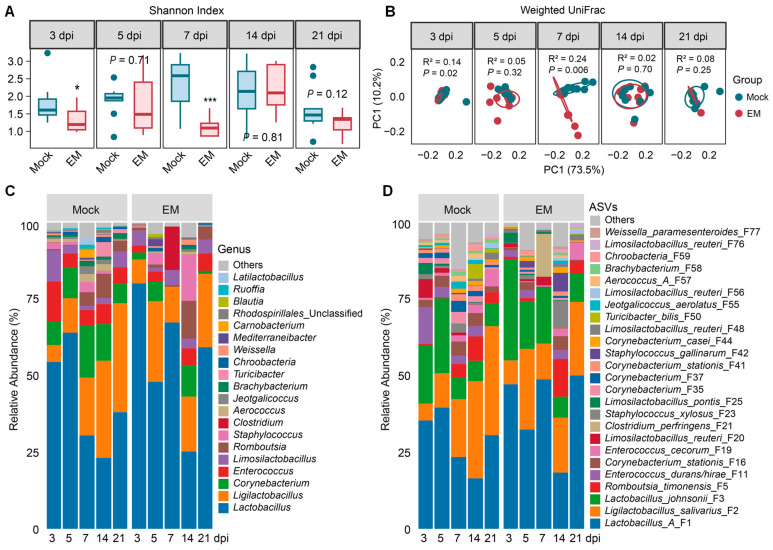
Ileal microbiota alternations in response to *Eimeria maxima* (EM) infection. (**A**) The Shannon index showing the α-diversity of the ileal microbiota in mock- and EM-infected chickens at different days post-infection (dpi). * *p* < 0.05 and *** *p* < 0.001, as determined by the Mann–Whitney U test at each time point. (**B**) The weighted UniFrac distances showing the β-diversity of the ileal microbiota in mock- and EM-infected chickens at different dpi. *p-* and R^2^-values were determined using permutational multivariate analysis of variance (PERMANOVA). (**C**) Changes in the relative abundance (%) of the top 20 ileal bacterial genera following infection. (**D**) Changes in the relative abundance (%) of the top 25 ileal bacterial amplicon sequence variants (ASVs) following infection.

In the ileum, EM-infected chicks exhibited significant reductions in α-diversity, as indicated by the Shannon index, at 3 dpi (*p* < 0.05) and 7 dpi (*p* < 0.001), which largely recovered by 14 dpi and 21 dpi (*p* > 0.1) (Figure 2A). Similarly, β-diversity, as measured using the weighted UniFrac distance, showed a shift in the microbiota composition at 3 dpi (*p* < 0.05) and 7 dpi (*p* < 0.01), followed by recovery at 14 and 21 dpi (Figure 2B).

Compositionally, Bacillota and Actinomycetota were the primary phyla in the ileum, accounting for 85.9–99.7% and 0.1–13.8% of total bacteria, respectively (Table S1). At the family level, *Lactobacillaceae*, *Corynebacteriaceae*, *Enterococcaceae*, *Staphylococcaceae*, and *Peptostreptococcaceae* accounted for approximately 90% of the total bacteria (Table S1). At the genus level, *Lactobacillus*, *Ligilactobacillus*, *Corynebacterium*, *Enterococcus*, and *Limosilactobacillus* were the five dominant genera, collectively accounting for 62.0–97.2% of the total bacteria (Figure 2C and Table S1). Two major *Lactobacillus* species included *Lactobacillus johnsonii* (F3) and Group A *Lactobacillus* (F1), with the latter consisting of highly related species such as *L. acidophilus*, *L. crispatus*, *L. gallinarum,* and *L. kitasatonis* that cannot be distinguished by sequencing the V3–V4 region of the bacterial 16S rRNA gene. *Ligilactobacillus* was primarily represented by *L. salivarius* (F2), and *Limosilactobacillus* was largely composed of *L. reuteri* (F20) and *L. pontis* (F25). *Corynebacterium stationis* (F16) was the main species within *Corynebacterium*, while *Enterococcus* was mainly composed of *E. durans/hirae* (F11) and *E. cecorum* (F19) (Figure 2D and Table S1).

**Figure 3:**
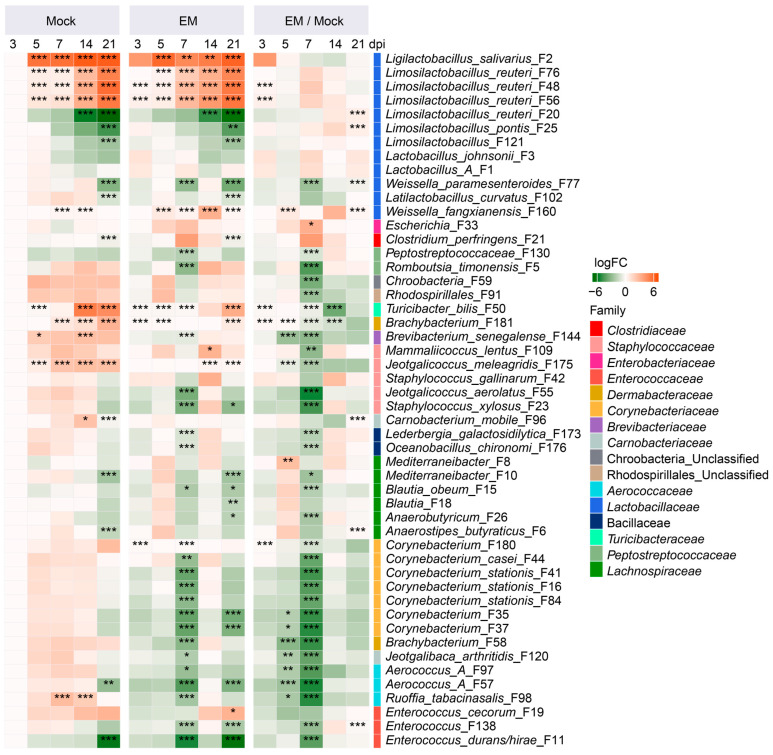
Dynamic changes in the differential enrichment of ileal bacteria in response to *Eimeria maxima* (EM) infection. The left and middle panels show log_2_ fold changes in the relative abundances of individual bacterial amplicon sequence variants (ASVs) compared to mock-infected animals at 3 days post-infection (dpi). The right panel illustrates the log_2_ fold change in each ASV in the EM group relative to the mock group at each time point. The family to which each ASV belongs is also indicated. Statistical differences were determined using ANCOM-BC [22], with * *p* < 0.05, ** *p* < 0.01, and *** *p* < 0.001.

To further reveal the differential abundance of the top 50 ileal bacterial ASVs in response to *Eimeria* infection, ANCOM-BC analysis [22] was conducted. Lactic acid bacteria (LAB) exhibited drastic age-dependent temporal changes in healthy, mock-infected chickens. For example, *L. salivarius* (F2) increased from 5.5% in day-13 (3 dpi) chickens to over 30% in day-24 (14 dpi) and day-31 (21 dpi) chickens, while *L. reuteri* (F20) and *L. pontis* (F25) were significantly reduced in day-31 chickens compared to younger birds (Figure 3 and Table S1). *L. johnsonii* (F3) also appeared to decline with age, while Group A *Lactobacillus* (F1) remained relatively stable in the ileum up to day 31 (21 dpi). Conversely, three less abundant strains of *L. reuteri* (F48, F56, and F76) were progressively enriched with age (Figure 3).

In response to EM infection, dominant LAB in the ileum, such as Group A *Lactobacillus* (F1), *L. salivarius* (F2), *L. johnsonii* (F3), and *L. reuteri* (F48, F56, and F76), had a tendency to enrich early at 3 dpi and then again during the peak of infection at 7 dpi (Figure 3). However, most other minor species of ileal bacteria experienced a gradual decline, with peak diminishment occurring at 7 dpi. For example, short-chain fatty acid (SCFA)-producing bacteria like *Romboutsia timonnensis* (F5), *Blautia obeum* (F15), and multiple *Corynebacterium* species (e.g., F16, F35, and F37) exhibited a significant decrease at 7 dpi. Opportunistic pathogens such as *Escherichia* (F33) were significantly enriched at 7 dpi, and *Clostridium perfringens* (F21) also numerically increased at 7 dpi (Figure 3). 

As animals recovered from coccidiosis, most bacteria appeared to be restored to healthy levels, although a few bacteria, such as *L. reuteri* (F20), *L. pontis* (F25), and *Anaerostipes butyraticus* (F6), showed statistically significant elevations at 21 dpi. Nevertheless, the magnitudes of their elevations were minimal, and statistical significance was achieved due to the absence of the bacteria in mock-infected birds in each case (Table S1), suggestive of the restoration of the ileal microbiota upon animal recovery from coccidiosis.

### 3.3. Cecal Microbiome Dynamics in Response to E. maxima Infection

**Figure 4:**
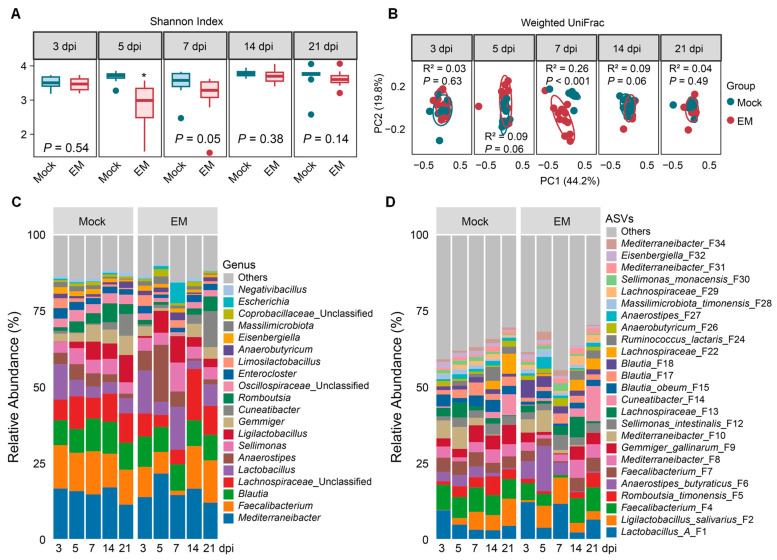
Cecal microbiota alternations in response to *Eimeria maxima* (EM) infection. (**A**) Shannon index showing the α-diversity of the cecal microbiota in mock- and EM-infected chickens at different days post-infection (dpi). * *p* < 0.05, as determined by the Mann–Whitney U test at each time point. (**B**) Weighted UniFrac distances showing the β-diversity of the cecal microbiota in mock- and EM-infected chickens at different dpi. *p-* and R^2^-values were determined using permutational multivariate analysis of variance (PERMANOVA). (**C**) Changes in the relative abundance (%) of the top 20 cecal bacterial genera following infection. (**D**) Changes in the relative abundance (%) of the top 25 cecal bacterial amplicon sequence variants (ASVs) following infection.

In the cecum, *EM* infection significantly decreased the Shannon index at 5 and 7 dpi (*p* ≤ 0.05), with no significant changes observed at other time points (Figure 4A). The most dramatic change in the weighted UniFrac distance was observed at 7 dpi (*p* < 0.05), with both mock- and EM-infected chickens showing highly similar β-diversity at 21 dpi (Figure 4B). Compositionally, the cecal microbiota was dominated by Bacillota, which accounted for approximately 99% of the total bacterial population (Table S2). At the family level, *Lachnospiraceae, Oscillospiraceae*, and *Lactobacillaceae* collectively represented about 90% of the total bacteria (Table S2). The top 20 genera comprised 84.6–90.1% of the total bacteria, with *Faecalibacterium*, *Mediterraneibacter*, and *Blautia* being the most abundant (Figure 4C and Table S2). Specifically, *Faecalibacterium* was dominated by two species (F4 and F7), while *Mediterraneibacter* was primarily represented by another two species (F8 and F10) (Figure 4D). *Blautia* was predominantly composed of *Blautia obeum* (F15) and two other species (F17 and F18). Notably, LAB was primarily represented by Group A *Lactobacillus* (F1) and *L. salivarius* (F2) (Figure 4D).

**Figure 5:**
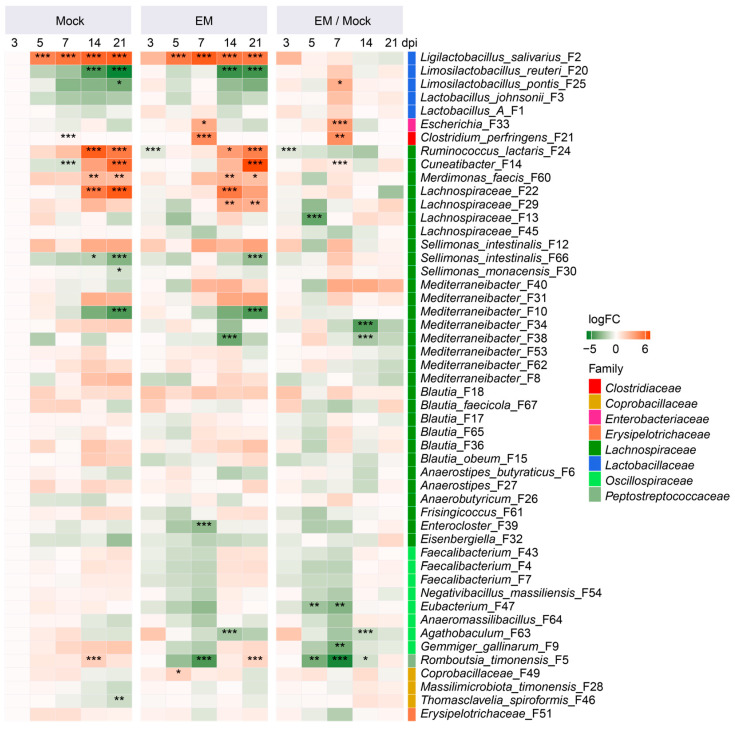
Dynamic changes in the differential enrichment of cecal bacteria in response to *Eimeria maxima* (EM) infection. The left and middle panels represent log_2_ fold changes in the relative abundances of individual bacterial amplicon sequence variants (ASVs) compared to mock-infected animals at 3 days post-infection (dpi). The right panel illustrates the log_2_ fold change in each ASV in the EM group relative to the mock group at each time point. The family to which each ASV belongs is also indicated. Statistical differences were determined using ANCOM-BC [22], with * *p* < 0.05, ** *p* < 0.01, and *** *p* < 0.001.

ANCOM-BC analysis [22] further revealed distinct temporal development patterns of cecal bacteria in healthy chickens (Figure 5). Similarly to the ileum, *L. salivarius* (F2) in the cecum was gradually enriched as the animals aged, while *L. reuteri* (F20) and *L. pontis* (F25) gradually declined. Group A *Lactobacillus* (F1) and *L. johnsonii* (F3) were largely unaffected. Additionally, several SCFA-producing *Lachnospiraceae* members, such as *Ruminococcus lactaris* (F24), *Cuneatibacter* (F14), *Merdimonas faecis* (F60), and *Lachnospiraceae* (F22), were significantly enriched in day-24 (14 dpi) and/or day-31 (21-dpi) chickens.

In response to EM infection, the cecal microbiota exhibited less pronounced changes compared to the ileal microbiota. LAB species were mostly enriched, particularly at 7 dpi. Opportunistic pathogens such as *Escherichia* (F33) and *C. perfringens* (F21) also experienced significant enrichment at 7 dpi. Multiple SCFA producers, including *R. timonensis* (F5), *Gemmiger gallinarum* (F9), and *Eubacterium* (F47), showed significant progressive declines, reaching their lowest levels at 7 dpi. Many other SCFA-producing bacteria, such as *Faecalibacterium* (F4, F7, and F43), also gradually diminished, bottoming out at 7 dpi, albeit in a less pronounced manner. Notably, all bacteria returned to normal levels by 21 dpi (Figure 5), consistent with the recovery of animals from coccidiosis.

## 4. Discussion

### 4.1. No Compensatory Weight Gain of Chickens After Recovery from Coccidiosis

Upon ingestion of sporulated oocysts by animals, EM invades and replicates inside intestinal epithelial cells to complete its life cycle [7,8,10]. This invasion causes damage to the intestinal mucosal epithelia between 5 and 7 dpi, leading to intestinal inflammation and impaired growth. Afterwards, clinical symptoms and intestinal barrier dysfunction gradually recover beyond 7 dpi. However, it remains unclear whether the intestinal microbiota is fully restored or permanently altered by coccidiosis even after recovery. To address this, we challenged day-10 broilers with EM to induce coccidiosis and analyzed the ileal and cecal microbiota compositions at multiple times up to 21 dpi (or 31 days of age). To minimize the risk of reinfection from oocysts shed by the birds, contaminated wood shavings were replaced with fresh ones at 8 dpi.

The chicken model of coccidiosis was evidently successful, as indicated by significant decreases in growth, feed intake, and feed efficiency during the first week, along with peak oocyst shedding and intestinal lesions at 7 dpi, consistent with the 7-day life cycle of EM. Infected birds showed rapid recovery, with weight gain, feed intake, and feed efficiency comparable to healthy, mock-infected birds during weeks 2 and 3 post-infection. Notably, EM infection had a long-term effect on body weight, with persistently reduced body weight up to 21 dpi and no compensatory growth. This finding aligns with an earlier meta-analysis [23], which indicated that full recovery in growth performance, including weight gain, feed intake, and feed conversion ratio, was not observed even at 30 dpi, the maximum time range evaluated. The lack of compensatory growth may be due to reduced feed intake in challenged birds, as they remain smaller than their healthy counterparts even after recovering from intestinal disorders [23].

### 4.2. Enrichment of Lactic Acid Bacteria in Response to E. maxima Infection

The small intestine is the primary site of EM infection [7]. During the peak of infection at 7 dpi, we observed significant alterations in the ileal microbiota. However, the cecal microbiota was also shifted by EM, although the change was less pronounced. EM-induced cecal microbiota alternations are likely due to the flow of reactive oxygen and nitrogen species, mucins, and cytoplasmic contents from damaged ileal epithelial cells to the cecum [24,25]. Both the α- and β-diversities of the ileal and cecal microbiota experienced the most drastic changes at 7 dpi, consistent with previous findings [13,14]. The reduction in the Shannon index of both the ileal and cecal microbiota at 7 dpi aligns with the EM-induced diminishment of various SCFA-producing bacteria and other bacterial species in both intestinal segments. This reduction in bacterial diversity is also observed in earlier studies on chicken coccidiosis [13,14], necrotic enteritis [17,26], and inflammatory bowel diseases [27].

LAB, such as *Lactobacillus*, *Ligilactobacillus*, and *Limosilactobacillus*, dominate the small intestine microbiota, accounting for 60–75% of the total bacterial population. These bacteria produce lactic acid and antimicrobial substances, inhibit inflammation, enhance intestinal barrier function, and aid in the repair of damaged intestinal mucosa, making them commonly used as probiotics [28,29]. *Lactobacillus* species are prevalent in the ileum of younger chicks but gradually decline, while *Ligilactobacillus* progressively increases and co-dominates the ileal microbiota with *Lactobacillus* by day 31. Major *Limosilactobacillus* species (F20) gradually decline, while a few minor *Limosilactobacillus* species (F48, F56, and F76) progressively increase in the ileum with age. Most of these LAB exhibit a similar temporal pattern in the cecum, although they only account for 10–15% of the total cecal bacteria. *Ligilactobacillus* gradually increases with a concurrent reduction in *Lactobacillus* and *Limosilactobacillus*. The temporal development of different LAB species may reflect a different intestinal environment and host physiological needs as animals age [29,30].

In response to EM-induced coccidiosis, *Lactobacillus* and *Limosilactobacillus* tend to increase, particularly at 7 dpi, while *Ligilactobacillus salivarius* tends to decline at 7 dpi and persists further in both the ileum and cecum, which is in agreement with earlier findings [13,14]. Additionally, some LAB species, such as two minor strains of *L. reuteri* (F48 and F56), are significantly enriched as early as 3 dpi, while a dominant *L. reuteri* strain (F20) and *L. pontis* are progressively enriched by EM and remain elevated even at 21 dpi. Different enrichment patterns of these LAB may suggest their potentially different roles in coccidiosis resistance. 

The rise in LAB during gut inflammation is likely due to their aerotolerant or facultative nature, which allows them to survive in the presence of reactive oxygen and nitrogen species generated by the host [31]. However, the pattern of LAB alterations varies among different intestinal inflammatory disorders. For instance, some studies report an increase in LAB in inflammatory bowel diseases, while others show contradictory results [31,32]. Additionally, most LAB species are significantly reduced in necrotic enteritis, especially in severely infected chickens [26]. These discrepancies are likely due to differences in the nature, severity, and duration of gut inflammation that the microbiota has been exposed to across various studies.

### 4.3. Diminishment of SCFA-Producing Bacteria in Response to E. maxima Infection

SCFAs, primarily acetate, propionate, and butyrate, are key bacterial fermentation products of dietary fibers in the large intestine, especially in the cecum of chickens [33]. SCFAs not only provide energy to intestinal epithelial cells, but also play a crucial role in maintaining intestinal health by regulating inflammation and enhancing intestinal barrier function [33]. In this study, dominant SCFA-producing bacteria such as *Faecalibacterium*, *Gemmiger gallinarum*, and *Romboutsia timonensis* were significantly suppressed by EM in the cecum. Additionally, the majority of SCFA producers, although minor in abundance, were also reduced in the EM-infected ileum. These results are consistent with previous studies on *Eimeria* infections [13,14,17], necrotic enteritis [26], and inflammatory bowel diseases [32]. Their decline is likely attributed to their obligate anaerobic nature, which results in a competitive disadvantage in an environment with high levels of reactive oxygen and nitrogen species generated by the host during gut inflammation [32]. The reduction in these SCFA producers is likely to exacerbate inflammation due to insufficient SCFA synthesis [32].

However, many SCFA producers commonly present on both sites were differentially regulated by EM. For example, *Mediterraneibacter* (F10), *Blautia* (F15), and *Anaerostipes butyraticus* (F6) were drastically suppressed by EM at 7 dpi in the ileum but showed no significant changes in the cecum. The reduced abundance of SCFA-producing bacteria is consistent with observations in other intestinal inflammatory disorders such as necrotic enteritis [26] and inflammatory bowel diseases [23].

Notably, multiple *Corynebacterium* species also significantly decreased at 7 dpi. Although *Corynebacterium* does not produce SCFAs, several *Corynebacterium* species exhibit antioxidant and antibacterial activities with probiotic potential [34]. Therefore, supplementation with SCFA-producing bacteria or *Corynebacterium* has the potential to reduce inflammation and support gut health, potentially mitigating the negative effects of the infection [33,34].

### 4.4. Transient Blooming of Pathobionts in Response to E. maxima Infection

In addition to the differential enrichment of LAB and SCFA producers, *Escherichia* significantly bloomed in both the ileum and cecum at 7 dpi of EM infection, aligning with previous studies on coccidiosis [14] and several other intestinal inflammatory diseases [23]. We believe this proliferation represents not only a proportional increase but also an absolute increase, as the total intestinal bacterial population remains relatively stable or even slightly increases in intestinal inflammatory disorders such as chicken necrotic enteritis [26] and human Crohn’s disease and ulcerative colitis [35]. The enrichment of *Escherichia* is likely due to the production of reactive oxygen and nitrogen species by host cells during intestinal inflammation, which subsequently diffuse into the lumen and react to form electron acceptors such as tetrathionate or nitrate, providing a fitness advantage for facultative bacteria like *Escherichia* to utilize as carbon sources [36].

Necrotic enteritis is caused by *C. perfringens*, but *Eimeria* infection is an important predisposing factor in both commercial broiler production and experimental infections [37,38]. *Eimeria* is commonly used in conjunction with *C. perfringens* to induce necrotic enteritis, and without prior *Eimeria* infection, chickens are notoriously resistant to experimentally induced necrotic enteritis [37,38]. In this study, although no exogenous *C. perfringens* was inoculated, we observed a bloom of commensal *C. perfringens* at the peak of infection in both the ileum and cecum. *Eimeria* infection appears to create a favorable environment for *C. perfringens* proliferation. Glycosylated mucins and cytoplasmic nutrients from damaged intestinal epithelial cells, as well as the ability to withstand O_2_ during intestinal inflammation, are among the major reasons for aerotolerant *C. perfringens* being able to thrive in the EM-inflamed gut [11]. 

### 4.5. Restoration of the Intestinal Microbiota After Recovery from Coccidiosis

External perturbations can alter the intestinal microbiota, but it often returns to its original state once the perturbation is removed. However, the microbiota may sometimes shift to an alternative healthy state depending on the nature of the perturbation [3]. In this study, we investigated the recovery of the ileal and cecal microbiota in chickens in response to coccidiosis. Our findings indicate that both the ileal and cecal microbiota are largely restored post-recovery, as evidenced by the lack of significant differences in α- and β-diversities between mock- and EM-infected chickens at 14 and 21 dpi. Specifically, the cecal microbiota showed no significant differences in major bacterial populations between the two groups by 21 dpi. In the ileum, major bacterial populations also returned to healthy levels by 14 dpi. Although some ileal bacteria exhibited statistically significant differences between mock- and EM-infected chickens, the magnitude of these differences was minimal, suggesting limited functional impact.

These results suggest that the intestinal microbiota of EM-recovered chickens is structurally and functionally similar to that of healthy chickens. This similarity likely contributes to the comparable weight gain, feed intake, and feed efficiency observed between the two groups of chickens beyond 14 dpi. It is noteworthy that no compensatory weight gain occurred in the chickens that had recovered clinically from coccidiosis at 21 dpi based on this study or 30 dpi according to a recent meta-analysis [23]. For compensatory weight gain to occur, the intestinal microbiota of recovered chickens would need to be more efficient in utilizing dietary components. However, the microbiota difference between the two groups of chickens was not observed in this study.

Despite the overall restoration, minor differences in bacterial composition persisted between healthy and EM-infected chickens at 21 dpi. Therefore, further extending the recovery period before analyzing microbiota changes could provide a clearer understanding of the long-term effects of coccidiosis on the intestinal microbiota. If these subtle differences persist, further investigation into the functional implications is warranted. Understanding the shifts in and restoration of the intestinal microbiota following coccidiosis could lead to new strategies for mitigating the disease.

## Data Availability

All results are available upon request. Bacterial 16S rRNA gene sequencing raw reads were deposited at the National Center for Biotechnology Information (NCBI) under accession number PRJNA1204488.

## Supplementary Materials

The following supporting information can be downloaded at: https://www.mdpi.com/article/10.3390/pathogens14010081/s1, Table S1. Differential enrichment of the ileal microbiota in response to *E. maxima* infection. Table S2. Differential enrichment of the cecal microbiota in response to *E. maxima* infection.
