# Supplementary material for: Is Intestinal Microbiota Fully Restored After Chickens Have Recovered from Coccidiosis?
Source: Pathogens. 2025 Jan 16;14(1):81. doi: 10.3390/pathogens14010081 (PMC11768824; doi:10.3390/pathogens14010081)
Supplement: Supplementary file 1 [file pathogens-14-00081-s001.zip › pathogens-3431832-supplementary.pdf]

Table S1. Differential enrichment of the ileal microbiota in response to *Eimeria maxima* infection

	3 dpi			5 dpi			7 dpi			14 dpi			21 dpi		
	Mock	EM	LFC	Mock	EM	LFC	Mock	EM	LFC	Mock	EM	LFC	Mock	EM	LFC
**Phylum**															
Bacillota	91.51	97.52	0.25	87.79	91.42	-0.06	80.32	98.45	0.37	83.93	87.98	0.81	90.19	98.14	0.33
Actinomycetota	8.25	2.4	-1.18	10.89	6.63	-1.48**	19.1	0.34	-4.37***	13.4	11.3	0.21	8.99	1.19	-1.89**
Cyanobacteriota	0.15	0.04	-0.24	1	1.07	-0.47	0.35	0.01	-3.71***	1.69	0.49	-0.42	0.61	0.45	-1.3
Total	99.91	99.96		99.68	99.12		99.77	98.8		99.02	99.77		99.79	99.78	
**Family**															
Lactobacillaceae	70.7	92.77	0.7	78.44	76.01	-0.28	53.33	83.81	1.17	57.75	48.29	-0.48	78.71	87.66	0.31
Corynebacteriaceae	5.96	1.98	-0.93	8.09	5.79	-1.45**	13.01	0.09	-4.63***	9.97	9.3	-0.54	5.48	0.92	-1.89
Enterococcaceae	13.09	2.28	-1.28	4.66	3.18	-0.67	5.48	0.4	-3.30***	6.52	5.62	-0.23	5.43	5.62	0.03
Staphylococcaceae	2.76	0.61	-0.69	1.68	4.26	-1.09	6.37	0.02	-4.66***	2.54	16.62	0.77	0.54	0.17	-1.14
Peptostreptococcaceae	0.96	1.02	-1.34	0.3	1.05	0.52	4.43	0.02	-4.14***	7.88	12.3	0.48	3.53	4.19	0.39
Clostridiaceae	0.16	0.01	-0.88	0.01	0.02	0.55	0.04	14.07	2.83	0.02	0.06	0.57	0.01	0	-0.77***
Mycobacteriaceae	1.64	0.31	-1.8	1.8	0.66	-2.08***	3.83	0.02	-4.61***	1.88	0.74	-1.39	0.75	0.05	-2.1
Lachnospiraceae	1.25	0.34	-0.77	0.42	5.26	1.59	1.61	0.12	-2.85***	0.74	0.72	0.23	0.11	0.02	-0.98
Aerococcaceae	1.16	0.29	-1.4	1.19	0.45	-2.32***	3.94	0	-5.71***	2.63	1.05	-1.21	0.32	0.09	-1.59
Dermabacteraceae	0.44	0.1	-0.88	0.66	0.02	-3.19***	1.42	0	-4.73***	0.99	0.9	-0.66	1.84	0.15	-2.07
Total	98.12	99.71		97.25	96.7		93.46	98.55		90.92	95.6		96.72	98.87	
**Genus**															
Lactobacillus	54.46	79.96	0.88	63.95	48.02	-0.82	30.59	67.27	1.34	23.3	25.36	-0.12	38.16	59.23	0.72
Ligilactobacillus	5.45	7.77	4.08	11.18	26.16	0.27	18.8	11.58	-0.85	31.58	17.84	-1.21	35.37	23.86	-0.23
Corynebacterium	7.6	2.29	-1.09	9.9	6.45	-1.53*	16.84	0.12	-5.12***	11.84	10.04	-0.63	6.23	0.98	-1.98
Enterococcus	13.09	2.27	-1.3	4.65	3.17	-0.65	5.07	0.4	-3.55***	6.5	5.6	-0.23	5.42	5.61	0.04
Limosilactobacillus	10.12	4.87	-0.46	2.53	1.25	-0.82	1.36	4.93	1.41	2.02	3.19	1.06	5.16	4.56	0.19
Romboutsia	0.4	0.91	-1.02	0.28	1.04	0.63	4.41	0.02	-4.46***	7.87	12.26	0.47	3.53	4.19	0.36
Staphylococcus	2.12	0.45	-0.55	0.94	3.73	-0.73	3.27	0.02	-4.31***	0.96	15.01	1.38	0.11	0.06	-0.7
Clostridium	0.08	0.01	-0.51	0	0.01	0.32	0.02	14.06	3.08	0.02	0.05	0.77	0.01	0	-0.48***
Aerococcus	1.08	0.24	-1.46	0.96	0.34	-2.46***	2.63	0	-5.60***	2.15	0.88	-1.15	0.12	0.05	-1.33
Jeotgalicoccus	0.59	0.13	-1.07	0.72	0.27	-1.91**	2.53	0	-5.47***	1.46	0.81	-0.81	0.38	0.06	-1.6
Brachybacterium	0.44	0.1	-0.89	0.66	0.02	-3.17***	1.42	0	-5.06***	0.99	0.9	-0.66	1.84	0.15	-2.1
Turicibacter	0	0	-0.13***	0	0	-0.06	0.02	0	-0.52***	4.71	0.03	-3.87***	1.31	0.26	-1.23
Chroobacteria	0.15	0.04	-0.16	1	1.07	-0.49	0.35	0.01	-3.60***	1.69	0.49	-1.27	0.61	0.45	-1.4
Weissella	0.63	0.16	-0.72	0.35	0.51	-0.44	0.36	0	-2.43***	0.75	1.8	-0.04	0	0	-0.24

	3 dpi			5 dpi			7 dpi			14 dpi			21 dpi		
	Mock	EM	LFC	Mock	EM	LFC	Mock	EM	LFC	Mock	EM	LFC	Mock	EM	LFC
Mediterraneibacter	0.4	0.1	-0.74	0.15	2.31	1.85*	0.62	0.04	-2.63**	0.18	0.3	0.75	0.02	0	-0.34
Carnobacterium	0.03	0	-0.51	0.04	0.02	0.03	2.59	0	-2.16	0.36	0.02	-1.87	0	0	0.13***
*Rhodospirillales*_Unclassified	0.05	0.01	-0.29	0.28	0.68	0.32	0.13	0.01	-2.67***	0.91	0.12	-1.82	0.18	0.21	-1.03
Blautia	0.34	0.1	-0.79	0.08	1.01	1.46	0.23	0.02	-2.91***	0.32	0.15	-0.4	0.03	0.01	-1.4
Ruoffia	0.07	0.01	-1.4	0.21	0.05	-2.33**	1.25	0	-5.10***	0.46	0.13	-1.65*	0.18	0.04	-1.34
Latilactobacillus	0.01	0	-0.65	0.12	0.04	-0.42	2.16	0	-2.28	0.06	0.01	-1.29	0	0	0.02
Mammaliicoccus	0.05	0.02	0.18	0.03	0.26	0.42	0.56	0	-2.95***	0.12	0.81	1.07	0.05	0.04	0.05
Jeotgalibaca	0.1	0.08	-0.38	0.25	0.05	-2.23***	0.87	0	-4.31***	0.2	0.14	-0.63	0.11	0.03	-0.83
Oceanobacillus	0.17	0.01	-1.16	0.31	0.15	-1.63	0.17	0	-2.84***	0.07	0.89	1.21	0	0.01	-0.01
Escherichia	0.02	0.01	-0.06	0.02	0.07	1.06	0	1.19	3.28**	0.03	0.07	0.21	0.02	0.01	0.01
Anaerostipes	0.19	0.05	-0.32	0.03	0.72	1.97*	0.33	0.02	-2.02	0.05	0.02	-0.34	0	0	0.08
Total	97.64	99.59		98.64	97.4		96.58	99.69		98.6	96.92		98.84	99.81	
**ASV**															
*Lactobacillus_A*_F1	35.47	47.22	0.83	39.64	32.52	-0.56	23.6	48.84	1.26	16.64	18.52	-0.12	30.69	50.1	0.69
*Ligilactobacillus_salivarius*_F2	5.45	7.77	4.05	11.18	26.16	0.29	18.8	11.58	-0.9	31.57	17.84	-1.17	35.36	23.86	-0.25
*Lactobacillus_johnsonii*_F3	18.99	32.74	1.03	24.31	15.49	-0.63	6.99	18.43	1.44	6.66	6.84	0	7.47	9.12	0.88
*Romboutsia_timonensis*_F5	0.4	0.91	-1.06	0.28	1.04	0.65	4.41	0.02	-4.51***	7.87	12.26	0.51	3.53	4.19	0.34
*Enterococcus_durans/hirae*_F11	12	2.11	-1.33	3.43	2.61	-0.5	2.65	0.06	-4.28***	3.39	2.81	-0.42	0.02	0.01	-0.24
*Corynebacterium_stationis*_F16	2.3	0.84	-0.85	3.61	2.55	-1.33	4.61	0.05	-4.37***	4.21	4.54	-0.46	1.92	0.36	-1.88
*Enterococcus_cecorum*_F19	0.65	0.09	-1.15	1.01	0.28	-2.55	2	0.34	-2.41	2.68	2.4	-0.45	5.4	5.6	0.1
*Staphylococcus_xylosus*_F23	1.65	0.16	-1.55	0.79	1.34	-1.41	2.79	0.01	-4.70***	0.72	8.95	1.24	0.07	0.03	-1.05
*Clostridium_perfringens*_F21	0.01	0	-0.39	0	0	0.08	0	13.94	3.96	0.01	0.04	1.09	0	0	0
*Limosilactobacillus_reuteri*_F20	6.09	1.4	-1.01	1.45	0.7	-0.56	0.92	2.82	-0.44	0.05	0.11	0.8	0	0.09	0.56***
*Corynebacterium*_F35	1.64	0.31	-1.85	1.8	0.66	-2.03*	3.83	0.02	-4.99***	1.88	0.74	-1.36	0.75	0.05	-2.14
*Corynebacterium*_F37	1.55	0.33	-1.5	1.77	0.62	-2.06*	3.6	0.02	-5.19***	1.71	0.69	-1.25	0.76	0.07	-1.92
*Staphylococcus_gallinarum*_F42	0.45	0.29	0.79	0.11	2.16	0.56	0.42	0.01	-2.15	0.14	6.05	2.59	0.04	0.03	0.45
*Corynebacterium_stationis*_F41	1.05	0.24	-1.26	1.35	0.73	-1.68	2.13	0.01	-4.77***	1.76	1.42	-0.7	1.04	0.2	-1.82
*Limosilactobacillus_pontis*_F25	3.61	3.34	0.37	0.68	0.4	-0.33	0.1	0.76	0.58	0.13	0.19	1.43	0	0.03	0.47***
*Corynebacterium_casei*_F44	0.5	0.45	0.1	0.92	1.43	-0.55	1.37	0.01	-3.97***	1.29	1.87	0.21	0.66	0.23	-1.22
*Limosilactobacillus_reuteri*_F48	0	0.01	0.25***	0.12	0.03	-0.21	0.1	0.47	2.06	0.83	1.27	0.27	2.25	1.89	-0.4
*Turicibacter_bilis*_F50	0	0	-0.17***	0	0	-0.04	0.02	0	-0.57***	4.71	0.03	-3.83***	1.31	0.26	-1.25
*Jeotgalicoccus_aerolatus*_F55	0.59	0.13	-1.1	0.7	0.27	-1.86	2.21	0	-5.44***	1.31	0.8	-0.52	0.12	0.06	-0.56
*Aerococcus_A*_F57	0.79	0.16	-2	0.74	0.25	-2.63***	1.79	0	-5.34***	1.52	0.69	-0.67	0.06	0.04	-0.52

	3 dpi			5 dpi			7 dpi			14 dpi			21 dpi		
	Mock	EM	LFC	Mock	EM	LFC	Mock	EM	LFC	Mock	EM	LFC	Mock	EM	LFC
*Brachybacterium*_F58	0.44	0.1	-0.93	0.66	0.02	-3.16***	1.36	0	-4.98***	0.95	0.9	-0.58	1.29	0.14	-1.93
*Chroobacteria*_F59	0.15	0.04	-0.19	0.96	1.05	-0.45	0.34	0.01	-3.57***	1.55	0.45	-1.18	0.59	0.44	-1.38
*Limosilactobacillus_reuteri*_F56	0	0.06	0.46***	0.11	0.03	-0.34	0.09	0.44	1.65	0.59	0.94	0.84	1.7	1.51	0.12
*Weissella_paramesenteroides*_F77	0.62	0.16	-0.76	0.34	0.5	-0.41	0.36	0	-2.69***	0.74	0.97	-0.5	0	0	-0.42***
*Limosilactobacillus_reuteri*_F76	0	0	0.01	0.06	0.02	-0.28	0.05	0.22	1.53	0.4	0.64	0.45	1.21	1.04	-0.21
*Corynebacterium_stationis*_F84	0.46	0.06	-1.38	0.34	0.18	-1.46	0.9	0	-4.02***	0.46	0.43	-0.74	0.38	0.04	-1.36
*Rhodospirillales*_F91	0.05	0.01	-0.32	0.28	0.68	0.34	0.13	0.01	-2.72***	0.91	0.12	-1.78	0.18	0.21	-1.05
*Carnobacterium_mobile*_F96	0	0	0.11	0	0.01	0.48	2.4	0	-1.19	0.23	0.01	-2.16	0	0	0.11***
*Mediterraneibacter*_F10	0.1	0.06	-0.68	0.11	1.45	1.13	0.5	0.01	-2.64*	0.06	0.06	0.33	0	0	0
*Aerococcus_A*_F97	0.29	0.08	-0.97	0.22	0.09	-2.20**	0.84	0	-4.45***	0.63	0.18	-2.69	0.06	0.01	-1.35
*Ruoffia_tabacinasalis*_F98	0.07	0.01	-1.44	0.21	0.05	-2.31*	1.25	0	-5.15***	0.46	0.13	-1.61	0.18	0.04	-1.36
*Latilactobacillus_curvatus*_F102	0.01	0	-0.68	0.12	0.04	-0.4	2.16	0	-2.33	0.06	0.01	-1.25	0	0	0
*Mammaliicoccus_lentus*_F109	0.05	0.02	0.15	0.03	0.26	0.45	0.56	0	-3.00**	0.12	0.81	1.11	0.05	0.04	0.04
*Escherichia*_F33	0.01	0.01	-0.07	0.02	0.07	1.19	0	1.19	3.20*	0.03	0.07	0.15	0.02	0.01	0.03
*Jeotgalibaca_arthritidis*_F120	0.1	0.07	-0.43	0.23	0.05	-2.22**	0.7	0	-4.05***	0.15	0.11	-0.53	0.07	0.02	-0.87
*Enterococcus*_F138	0.19	0.04	-0.69	0.11	0.1	-1.21	0.17	0	-2.86***	0.31	0.17	0.94	0	0	-0.13***
*Anaerostipes_butyraticus*_F6	0.16	0.04	-0.08	0.03	0.43	1.93	0.29	0.01	-2.15	0.03	0.02	-0.42	0	0	0.11***
*Brevibacterium_senegalense*_F144	0.01	0	-0.38	0.11	0	-2.72***	0.22	0	-3.01***	0.15	0.04	-1.81	0.48	0.02	-2
*Limosilactobacillus*_F121	0.35	0.05	-0.57	0.09	0.07	-0.43	0.08	0.21	0.13	0.01	0.02	0.34	0	0	0
*Blautia_obeum*_F15	0.19	0.03	-1	0.02	0.31	1.72	0.06	0	-2.00***	0.16	0.04	-0.64	0.01	0	-1.02
*Weissella_fangxianensis*_F160	0	0	0.01	0	0.01	0.75***	0	0	0.09	0	0.83	2.93	0	0	0.20***
*Blautia*_F18	0.06	0.03	-0.49	0.02	0.4	1.55	0.07	0	-1.73	0.07	0.03	-0.4	0	0	-0.2
*Peptostreptococcaceae*_F130	0.56	0.12	-0.38	0.01	0	-0.28	0.02	0	-1.10***	0.01	0.03	1	0	0	-0.05
*Mediterraneibacter*_F8	0.04	0.02	-0.07	0.01	0.42	2.54**	0.04	0.01	-0.91	0.04	0.09	1.32	0.01	0	-0.25
*Lederbergia_galactosidilytica*_F173	0.23	0	-0.89	0.05	0.02	-1.11	0.06	0	-1.97***	0.03	0.32	1	0	0.01	0.57
*Oceanobacillus_chironomi*_F176	0.08	0	-0.85	0.07	0.02	-1.31	0.08	0	-1.87***	0.02	0.4	1.13	0	0	0.14
*Jeotgalicoccus_meleagridis*_F175	0	0	0.01	0.02	0	-0.70***	0.33	0	-2.27***	0.15	0.01	-2.29	0.18	0	-2.26
*Corynebacterium*_F180	0	0	-0.10***	0	0	-0.48	0.02	0	-1.15***	0.01	0	-0.75	0.62	0	-2.39
*Brachybacterium*_F181	0	0	0.13***	0	0	0.07***	0.06	0	-0.83***	0.05	0	-1.23***	0.54	0.01	-2.28
*Anaerobutyricum*_F26	0.1	0.02	-0.66	0.03	0.26	1.1	0.11	0.01	-2.40***	0.02	0.04	0.67	0	0	-0.2
Total	97.51	99.53		98.15	95.78		95.59	99.51		97.48	95.93		99.02	99.76	

Note: Average relative abundances (%) of the top ileal bacterial phyla, families, genera, and amplicon sequence variants (ASVs) in the ileum across five different days post-infection (dpi) with *E. maxima* (EM) are shown. *$p < 0.05$, **$p < 0.01$, ***$p < 0.001$, as determined by ANCOM-BC analysis, which generated $\log_2$ fold change (LFC) as well.

Table S2. Differential enrichment of the cecal microbiota in response to *Eimeria maxima* infection

	3 dpi			5 dpi			7 dpi			14 dpi			21 dpi		
	Mock	EM	LFC	Mock	EM	LFC	Mock	EM	LFC	Mock	EM	LFC	Mock	EM	LFC
**Phylum**															
Bacillota	99.27	99.79	-0.01	99.56	98.87	-0.11	99.68	90.94	-0.03	99.41	99.79	-0.01	99.79	99.91	0.06
Pseudomonadota	0.49	0.1	-0.18	0.33	0.95	0.45	0.14	7.01	3.73***	0.49	0.1	-0.64	0.06	0.04	-0.03
Actinomycetota	0.24	0.11	-0.38	0.11	0.17	0	0.18	2.03	2.10***	0.1	0.09	-0.03	0.15	0.04	-1.14***
Total	100	100		100	99.99		100	99.98		100	99.98		100	99.99	
**Family**															
Lachnospiraceae	49.24	51.9	0.03	53.9	63.42	-0.13	50.58	50.25	0.19	54.77	57.44	0.02	50.1	55.76	0.29
Oscillospiraceae	27.79	22.94	-0.26	26.42	12.43	-1.20*	29.81	7.04	-1.71***	24.64	27.48	0.04	28.23	24.78	-0.09
Lactobacillaceae	15.01	18.2	0.71	8.77	12.25	-0.04	9.94	25.2	1.34	9.42	6.19	-0.38	14.27	10.75	-0.03
Coprobacillaceae	3.16	3.06	0.1	3.54	6.39	0.3	3	2.41	-0.31	1.91	3.72	0.75	1.22	1.65	0.45
Peptostreptococcaceae	1.27	1.47	-0.05	3.65	0.89	-3.67***	3.37	0.03	-5.35***	6.34	2.07	-1.27***	3.24	4.77	0.6
Enterobacteriaceae	0.45	0.09	-0.24	0.3	0.95	0.47	0.12	6.71	3.74***	0.48	0.08	-0.72	0.05	0.04	0.07
Erysipelotrichaceae	1.35	0.99	-0.42	1.31	1.38	-0.65	1.67	0.36	-2.04*	0.74	0.68	-0.06	0.56	0.62	0.1
Clostridiaceae	0.42	0.27	-0.5	0.31	0.11	-1.3	0.22	4.38	1.13	0.05	0.05	0.2	0.02	0.03	0.28
Enterococcaceae	0.45	0.14	-1.25	0.13	1.73	-0.44	0.16	0.83	0.29	0.21	0.05	-1.22	0.12	0.06	-0.72
Bacillaceae	0.12	0.34	-0.2	0.88	0.09	-2.13	0.56	0.16	-1.18	0.29	0.87	0.77	0.45	0.24	-1.27
Total	99.26	99.4		99.21	99.64		99.43	97.37		98.85	98.63		98.26	98.7	
**Genus**															
Mediterraneibacter	16.77	13.98	-0.25	15.91	21.7	0.1	14.86	14.62	0.46	17.17	16.73	-0.15	11.5	12.14	0.12
Faecalibacterium	14.23	9.92	-0.76	12.59	7.1	-1.84	14.14	1.42	-1.52	11.03	13.98	0.15	11.44	13.88	0.39
Blautia	8.2	9.94	0.2	7.83	8.19	-0.4	10.75	8.68	0.53	10.48	8.45	-0.24	8.86	8.35	0.12
*Lachnospiraceae*_Unclassified	6.77	7.58	0.11	10.73	3.93	-1.61*	6.86	4.79	-0.02	9.26	16.87	0.59	9.68	9.49	0.2
Lactobacillus	11.63	14.03	0.85	5.42	4.3	-0.3	3.6	14.12	1.69	3.72	2.72	0.09	4.98	7.08	0.47
Anaerostipes	3.66	6.46	0.27	5.12	18.65	0.71	4.38	5	0.03	2.6	0.67	-1.47*	1.44	1.65	0.28
Sellimonas	3.54	4.24	0.18	5.18	3.97	-0.57	4.47	9.5	1.28	5.06	3.84	-0.31	3.74	4	0.27
Ligilactobacillus	0.09	0.63	2.83	2.15	7.17	0.37	5.88	8.6	0.83	4.99	3.15	-0.56	8.89	2.78	-0.81
Gemmiger	2.66	3.17	0.27	2.76	0.95	-2.08	5.51	0.32	-3.66***	4.56	4.39	-0.94	6.33	3.72	-0.69
Cuneatibacter	0.91	0.65	0.01	0.33	1.1	-0.2	0.45	0.1	-1.35	2.42	3.01	-0.26	7.15	11.86	0.98
Romboutsia	1.15	1.46	0.01	3.52	0.86	-3.63***	3.34	0.03	-5.34***	6.18	2.02	-1.25*	3.22	4.77	0.61
Oscillospiraceae_Unclassified	2.86	2.13	-0.39	3.76	1.34	-1.33***	3	0.81	-1.36**	3.3	2.06	-0.39	3.1	2.09	-0.37
Enterocloster	3.41	2.43	-0.15	2.64	1.12	-1.61	1.96	1.51	-1.28	2.19	2.37	0.04	1.8	2.03	0.29
Limosilactobacillus	3.25	3.54	0.57	1.19	0.79	-0.34	0.46	2.47	2.61	0.7	0.31	0.24	0.4	0.89	1.09

	3 dpi			5 dpi			7 dpi			14 dpi			21 dpi		
	Mock	EM	LFC	Mock	EM	LFC	Mock	EM	LFC	Mock	EM	LFC	Mock	EM	LFC
Anaerobutyricum	1.49	1.45	-0.05	0.73	1.5	0.29	0.85	2.53	0.98	0.76	0.48	-0.54	2.1	1.27	-0.53
Eisenbergiella	2.16	1.67	-0.36	1.56	1.19	-0.88	1.66	1.33	-0.25	1.18	0.96	-0.46	0.66	0.85	0.24
Massilimicrobiota	1.67	1.45	0.19	1.68	2.46	-0.09	1.24	0.93	-0.46	0.58	1.09	0.78	0.31	0.45	0.48
Coprobacillaceae_Unclassified	0.63	0.75	0.44	0.98	2.42	0.45	1.14	0.8	-0.7	0.82	1.52	0.49	0.67	0.71	0.22
Escherichia	0.45	0.09	-0.25	0.3	0.95	0.48	0.12	6.71	3.74***	0.48	0.08	-0.72	0.05	0.04	0.08
Negativibacillus	0.89	1.09	0.13	1.12	0.45	-1.58	1.07	0.35	-1.77*	0.91	1.24	0.13	1.29	0.63	-0.69
Eubacterium	1.39	1.09	-0.61	1.2	0.4	-2.39***	1.16	0.25	-2.35	0.96	1.04	-0.06	0.84	0.63	-0.12
Thomasclavelia	0.86	0.86	-0.15	0.88	1.51	-0.09	0.62	0.68	0.13	0.51	1.11	1	0.24	0.49	0.77
*Erysipelotrichaceae*_Unclassified	0.87	0.83	0.35	1.1	1.2	-0.67	1.39	0.27	-2.04	0.63	0.62	-0.05	0.46	0.54	0.17
Agathobaculum	1.41	1.84	-0.12	1.31	0.32	-1.43	1.09	0.43	-1.95	0.22	0.41	0.39	0.38	0.26	-0.48
Oliverpabstia	0.19	1.13	1.95	0.58	0.86	-1.43	2.19	0.15	-2.12	0.57	0.11	-0.85	0.2	0.61	1.66
Total	91.14	92.41		90.57	94.43		92.19	86.4		91.28	89.23		89.73	91.21	
**ASV**															
*Lactobacillus_A*_F1	9.67	12.33	1.03	4.95	3.92	-0.31	3.26	11.78	1.59	3.06	2.37	0.03	4.54	6.57	0.42
*Faecalibacterium*_F4	8.24	5.55	-0.69	6.93	3.93	-1.78	8.01	0.81	-1.82	6.57	8.08	0.07	6.47	7.82	0.37
*Ligilactobacillus_salivarius*_F2	0.09	0.63	2.82	2.15	7.17	0.37	5.88	8.6	0.82	4.99	3.15	-0.56	8.89	2.78	-0.81
*Anaerostipes_butyraticus*_F6	3.12	5.79	0.22	3.67	14.88	0.43	3.54	4.06	-0.33	1.12	0.29	-1.49	0.46	0.44	-0.08
*Faecalibacterium*_F7	4.62	3.41	-0.93	4.46	2.44	-1.5	4.68	0.45	-1.53	3.3	4.37	0.27	3.76	4.51	0.31
*Gemmiger_gallinarum*_F9	2.66	3.17	0.27	2.76	0.95	-2.07	5.51	0.32	-3.66**	4.56	4.39	-0.94	6.33	3.72	-0.69
*Mediterraneibacter*_F8	3.69	2.78	-1.46	1.35	1.2	-0.67	3.3	2.25	-0.07	4.41	5.95	-0.84	4	4.44	-1.69
*Sellimonas_intestinalis*_F12	1.37	2.35	1.99	3.09	2.13	-2.23	2.47	4.98	2.64	3.64	2.62	-0.58	2.88	3.15	0.28
*Mediterraneibacter*_F10	5.88	4.37	0.6	7.22	7.12	-0.71	2.48	1.13	-0.56	0.29	0.37	-0.09	0	0	0
*Romboutsia_timonensis*_F5	1.15	1.46	0.01	3.52	0.86	-3.62**	3.34	0.03	-5.35***	6.18	2.02	-1.25*	3.22	4.77	0.61
*Lachnospiraceae*_F13	2.76	2.17	-0.48	4.9	0.92	-4.01***	1.34	2.82	0.07	1.76	6.73	1.31	0.25	0.67	0.98
*Cuneatibacter*_F14	0.46	0.27	-0.13	0	0.89	1.03	0	0.03	0.73***	2.03	2.73	-0.91	6.91	11.54	1.02
*Blautia*_F17	1.46	1.3	-0.4	2.79	1.33	-1.05	4.26	3.32	0.55	1.94	2.27	0.09	3.04	1.79	-0.64
*Blautia_obeum*_F15	3.91	0.99	-1.42	1.49	2.37	-0.85	3.52	0.82	-0.35	3.97	1.87	-1.32	2.76	2.04	-0.25
*Blautia*_F18	1.22	5.82	2.3	1.35	3.53	-0.4	1.74	2.34	1.67	1.52	1.7	0.54	0.93	1.63	0.56
*Lachnospiraceae*_F22	0.28	0.02	-0.32	0.07	0.7	0.62	0	0.06	1.23	2.76	3.56	0.02	6.52	3.57	-2.6
*Ruminococcus_lactaris*_F24	0.04	0	-0.89***	1.39	0.34	-1.09	1.11	0.12	-1.67	4.54	2.93	-2.32	2.32	2.6	-0.18
*Anaerobutyricum*_F26	1.49	1.45	-0.06	0.72	1.5	0.39	0.75	2.53	1.54	0.5	0.48	0.19	1.91	1.25	0.02
*Anaerostipes*_F27	0.54	0.67	0.4	1.45	3.77	-0.96	0.84	0.8	-0.45	1.48	0.38	-1.46	0.98	1.21	0.35
*Lachnospiraceae*_F29	0.17	1.35	-0.37	1.46	0.03	-2.94	1.33	0.59	-0.41	1.6	2.88	0.59	0.55	2.16	1.57

	3 dpi			5 dpi			7 dpi			14 dpi			21 dpi		
	Mock	EM	LFC	Mock	EM	LFC	Mock	EM	LFC	Mock	EM	LFC	Mock	EM	LFC
*Massilimicrobiota_timonensis*_F28	1.67	1.45	0.19	1.68	2.46	-0.09	1.24	0.93	-0.47	0.58	1.09	0.78	0.31	0.45	0.48
*Mediterraneibacter*_F31	0.63	0.8	0.3	1.14	0.61	-0.96	0.54	1.59	1.72	1.69	1.91	0.3	0.97	1.69	0.79
*Sellimonas_monacensis*_F30	1.2	1.09	-0.2	1.36	1.27	-0.2	1.38	2.56	1.02	0.85	0.7	-0.16	0.41	0.5	0.43
*Eisenbergiella*_F32	2.02	1.53	-0.74	1.38	1.17	-0.09	1.37	0.84	-0.8	1.08	0.81	-0.89	0.35	0.76	1.44
*Mediterraneibacter*_F34	0.86	0.7	-0.57	0.66	2.72	1.04	1.26	1.85	-1.45	1.41	0.05	-4.49***	0.83	0.38	-1.96
*Blautia*_F36	0.6	1.23	0.54	1.27	0.46	-1.79	0.37	0.79	1.23	1.7	1.29	-0.63	1.02	1.85	0.8
*Mediterraneibacter*_F38	1.6	2.25	0.03	0.51	1.98	1.46	2.2	0.64	-0.72	0.19	0	-2.38***	0.42	0.14	-1.65
*Enterocloster*_F39	2.07	1.31	-0.3	1.52	0.76	-2.13	0.96	0.22	-2.02	1.01	0.86	-0.13	0.67	0.8	0.44
*Mediterraneibacter*_F40	0.56	0.48	0.15	0.88	0.02	-2.13	0.21	3.61	3.44	0.4	2.16	3.45	0.05	0.99	2.78
*Escherichia*_F33	0.44	0.09	-0.14	0.27	0.78	0.73	0.1	6.49	4.01***	0.47	0.07	-1.07	0.05	0.04	0.02
*Lactobacillus_johnsonii*_F3	1.97	1.7	0.14	0.47	0.38	-0.12	0.34	2.34	2.44	0.66	0.36	0.32	0.44	0.5	0.73
*Faecalibacterium*_F43	1.12	0.73	-0.56	0.95	0.51	-0.98	1.15	0.13	-1.44	0.87	1.28	0.43	1	1.22	-0.06
*Lachnospiraceae*_F45	1.13	1.44	0.41	0.99	0.6	-0.92	1.01	0.3	-1.97	0.8	0.61	-0.69	0.69	0.67	0.11
*Thomasclavelia_spiroformis*_F46	0.85	0.86	-0.16	0.88	1.49	-0.13	0.61	0.64	0.1	0.51	1.11	1.01	0.24	0.49	0.76
*Eubacterium*_F47	0.95	0.79	-0.9	1.07	0.39	-2.62**	1.07	0.15	-3.28**	0.95	0.94	-0.16	0.84	0.63	-0.12
*Limosilactobacillus_reuteri*_F20	1.9	1.59	0.18	0.49	0.53	0.4	0.4	1.69	2.05	0.11	0.05	-0.41	0.01	0.12	0.61
*Erysipelotrichaceae*_F51	0.75	0.71	0.34	0.94	1.05	-0.66	1.23	0.23	-2.05	0.54	0.53	-0.14	0.39	0.46	0.2
*Coprobacillaceae*_F49	0.32	0.47	0.6	0.54	2.17	0.96	0.72	0.73	-0.31	0.3	0.82	1.25	0.23	0.15	-0.32
*Negativibacillus_massiliensis*_F54	0.59	0.68	0.12	0.82	0.33	-1.53	0.74	0.23	-2.17	0.62	0.86	0.19	0.9	0.45	-0.65
*Mediterraneibacter*_F53	0.34	0.55	-0.16	0.44	2.45	0.22	0.56	0.27	0.33	0.81	0.33	-0.38	0.11	0.02	-0.83
*Frisingicoccus*_F61	0.4	0.24	-0.9	0.39	0.17	-2.21	0.37	0.44	-0.55	0.83	0.66	-0.33	0.93	0.78	-0.12
*Merdimonas_faecis*_F60	0.17	0.29	0.82	0.59	0.12	-1.98	0.3	0.77	0.93	0.63	0.96	0.23	0.67	0.58	-0.08
*Mediterraneibacter*_F62	0.23	0.39	-0.13	0.36	1.87	1.02	0.64	0.19	-0.62	0.64	0.25	-0.88	0.16	0.01	-1.46
*Limosilactobacillus_pontis*_F25	1.29	1.81	0.66	0.61	0.22	-0.6	0.03	0.46	3.24*	0.2	0.02	0.37	0.01	0.05	0.25
*Agathobaculum*_F63	0.84	1.75	2.19	0.99	0.15	-0.74	0.68	0.25	-2.44	0.03	0	-1.47***	0.06	0.01	-0.98
*Anaeromassilibacillus*_F64	0.98	0.72	0.25	0.53	0.36	-1.15	0.92	0.19	-2.42	0.22	0.53	0.64	0.11	0.18	0.47
*Clostridium_perfringens*_F21	0	0	0.01	0	0	0.01	0	4.17	4.51**	0	0	0	0	0	0
*Blautia*_F65	0.52	0.21	-0.57	0.55	0.22	-1.43	0.35	0.94	1.29	0.63	0.47	-0.42	0.29	0.37	0.44
Total	79.72	83.04		78.8	86.06		79.93	83		79.46	78.18		79.04	81.42	

Note: Average relative abundances (%) of the top cecal bacterial phyla, families, genera, and amplicon sequence variants (ASVs) in the ileum across five different days post-infection (dpi) with *E. maxima* (EM) are shown. *$p < 0.05$, **$p < 0.01$, ***$p < 0.001$, as determined by ANCOM-BC analysis, which generated $\log_2$ fold change (LFC) as well.
